# Changes in Facial Profile after Modified Anterior Maxillary Subapical Osteotomy

**DOI:** 10.3390/jpm12030508

**Published:** 2022-03-21

**Authors:** Chun-Ming Chen, Szu-Ting Chou, Shih-Chieh Chen, Chin-Yun Pan, Kun-Jung Hsu, Yu-Chuan Tseng

**Affiliations:** 1 Department of Oral and Maxillofacial Surgery, Kaohsiung Medical University, Kaohsiung 80708, Taiwan; komschen@gmail.com; 2 School of Dentistry, College of Dental Medicine, Kaohsiung Medical University, Kaohsiung 80708, Taiwan; stchou@kmu.edu.tw (S.-T.C.); kjhsu@kmu.edu.tw (K.-J.H.); 3 Department of Orthodontics, Kaohsiung Medical University Hospital, Kaohsiung 80756, Taiwan; anakin0430@gmail.com (S.-C.C.); spig.pan6363@gmail.com (C.-Y.P.); 4 Department of Dentistry, Kaohsiung Medical University Hospital, Kaohsiung 80756, Taiwan

**Keywords:** bimaxillary protrusion, anterior maxillary subapical osteotomy, facial profile, soft–hard tissue ratio, Wassmund technique, Wunderer technique

## Abstract

(1) Background: This study explored the effects of modified anterior maxillary subapical osteotomy (AMSO) on facial profile changes in patients with bimaxillary protrusion. (2) Methods: Cephalograms of patients were collected preoperatively and over 2 months postoperatively. The following landmarks were recorded: pronasale (Prn), subnasale (Sn), labrale superius (Ls), anterior nasal spine (ANS), and incisor superius (Is). The following distances and angles were measured: ANS–Prn, ANS–Sn, ANS–Ls, Is–Sn, Is–Ls, SNA angle, and nasolabial (NLA) angle. (3) Results: Is and ANS were significantly retracted by 7.3 and 2.3 mm, respectively. Soft tissue landmarks (Prn, Sn, and Ls) were significantly retracted (1.2, 1.6, 4.4 mm, respectively). Postoperative changes in soft/hard tissue ratios were 0.54, 0.72, 0.31, and 0.60 for Prn/ANS, Sn/ANS, ANS/Is, and Ls/Is, respectively. The NLA angle was increased significantly by 7.1°. (4) Conclusions: The horizontal soft/hard tissue ratios of Sn/Is, ANS/Is, and Ls/Is were 0.22, 0.31, and 0.60, respectively. The NLA angle was increased significantly by 7.1°. The modified AMSO provides an increased blood supply, allows for direct vision, and results in fewer complications than other AMSO methods.

## 1. Introduction

Bimaxillary protrusion (BiP), characterized by protruding upper and lower incisors and lips, results in a convex profile. Moreover, patients often have lip incompetence, a gummy smile, and mentalis muscle strain [1,2]. Anterior maxillary subapical osteotomy (AMSO) is an alternative orthognathic technique in the treatment of BiP [3,4,5]. BiP is a relatively common anomaly in the Asian population with different degrees of severity. Clinically, BiP is treated according to the severity of the deformity, and correction methods can be orthodontic or combined with orthognathic surgery.

Recently, due to advances in surgical theories and technologies, as well as the development of hypotensive anesthesia and operative equipment, surgical correction of BiP has become an alternative treatment. Some patients also opt for combined surgical treatment to improve the facial profile and reduce treatment time. After its introduction by Cohn-Stock [6] in 1921, AMSO was modified by Wassmund [7]. Later, Cupar [8] and Wunderer [9] modified the incision design and osteotomy approach of AMSO for soft tissues and bones to maintain a clear surgical field and ensure the sufficient blood supply to the anterior segment of the maxilla. However, AMSO was not popularized until 1969, when Bell [10] conducted an animal study to observe microvascular circulation. The experimental results confirmed that even if the palatal or labiobuccal soft tissue pedicel was retained on one side, blood supply would have immediately recirculated to the anterior maxillary segment and root regions of the teeth after the reattachment of the reflected mucoperiosteal flap. Notably, healing occurred in the contact area of the two bone segments within 6 weeks.

The literature [11,12,13,14,15] indicates that postoperative complications of AMSO include palatal tear, dental hypersensitivity, oro–antral–nasal fistula, and bone necrosis. Gunaseelan et al. [12] evaluated intraoperative and perioperative complications in 103 patients undergoing AMSO by the Cupar [8] method. Examining soft tissue injuries and vascular considerations, they reported that 27 patients (26.2%) experienced complications of varying severities: 43.3% were soft tissue-related, and 36.6% were attributable to dental causes. Palatal mucosal tear was the most common complication (11/103 = 10.7%). Regarding severe complications of AMSO, necrosis of the anterior maxilla was reported in the literature [14,15]. Specifically, the anterior maxillary segment sloughed postoperatively due to the severing of the blood supply to both the labial and palatal flaps. Therefore, surgeons must take great care in maintaining the integrity of blood supply to these areas.

Surgeons must aim to maintain a sufficient blood supply and implement well-controlled approaches for AMSO. For improving the intervention and prognosis, Perrotti et al. [16] used the multiplane three-dimensional (3D) total face cephalometry protocol to program and define the orthodontic treatment and orthognathic surgery. They found that 3D technology (3D imaging, 3D surgical stimulation software, and 3D printing) are sharpening in the pre-surgical planning, perioperative intervention, and prognosis. In the present study, we developed a modified AMSO method and investigated postoperative changes in soft and hard tissue.

## 2. Materials and Methods

**Figure 1:**
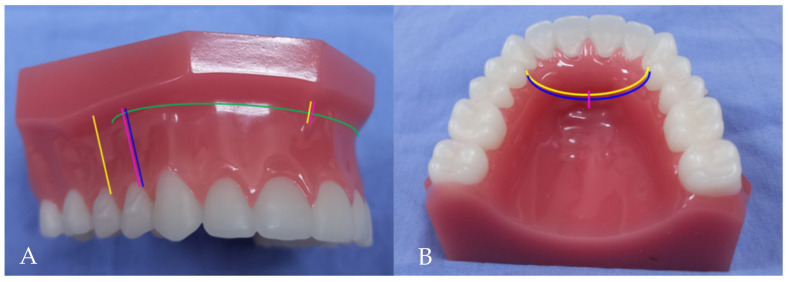
Four different anterior maxillary subapical osteotomies (AMSO). Yellow line: present study (Modified AMSO); Green line: Cupar method; Blue line: Wunderer method; Pink line: Wassmund method. (**A**) Vestibular incisions (**B**) Palatal mucosal incisions.

Patients with BiP treated at the Department of Oral and Maxillofacial Surgery of Kaohsiung Medical University were recruited if they (1) had no significant lip deformation before surgery and (2) had no history of abnormal facial development or facial trauma. Thirty-three patients were enrolled, aged 16 to 43 years, with a mean age of 27.5 years. Each participant underwent modified AMSO (Yellow line in the Figure 1A,B).

**Figure 2:**
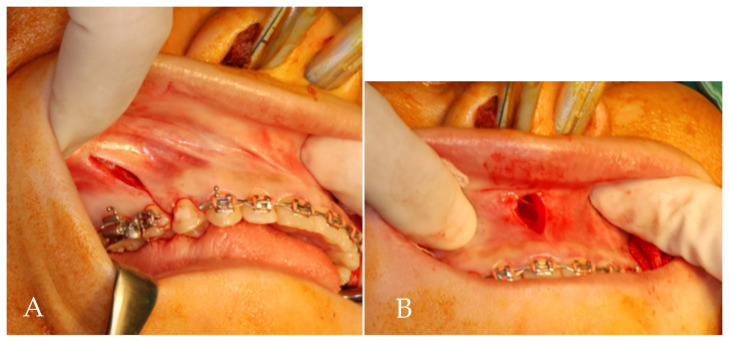
(**A**) Vestibular incision of modified AMSO: a vertical releasing incision from the posterior to distal interdental papilla of the first premolar, extending to the vestibular depth. (**B**) A vertical incision is made through the mucoperiosteum overlying the anterior nasal spine and carried inferiorly 5 mm.

**Figure 3:**
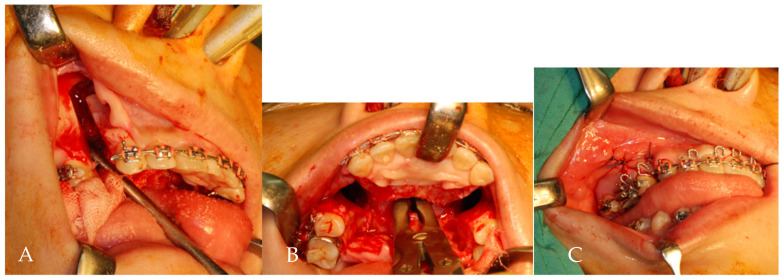
(**A**) Buccal ostectomy of lateral maxilla through retracted wound margins. (**B**) An anterior curving transpalatal ostectomy was performed to connect bilateral buccal ostectomy sites. A rongeur forcep was used to remove a small portion of nasal septum. (**C**) A predetermined palatal acrylic splint fixed with anterior and posterior teeth by circumdental wires.

Modified AMSO was performed by making a vertical releasing incision from the posterior to distal interdental papilla of the first premolar, extending to the vestibular depth (Figure 2A). The mucoperiosteal flap was raised from 5 mm above the apex of the first premolar to the lateral rim of the piriform aperture using a subperiosteal tunneling technique. A vertical incision was made through the mucoperiosteum overlying the anterior nasal spine (ANS) and extended 5 mm inferiorly (Figure 2B). Through subperiosteal tunneling beneath the ANS, the mucoperiosteal flaps overlying the medial rim of the piriform aperture and nasal floor were reflected. An arching palatal incision was made from the anterior to the medial interdental papilla of the first premolar and continued to the contralateral side. The palatal flap was reflected posteriorly to the first molar area. Under protection of nasal mucosa, subapical osteotomy (5 mm above tooth apex) and bone removal were performed in the region of extracted tooth and then oblique to infero-lateral portion of piriform aperture (Figure 3A).

Under direct vision, an anterior curving transpalatal ostectomy was performed to connect bilateral buccal ostectomy sites (Figure 3B). A small portion of nasal septum was removed by rongeur forcep. Subsequently, the anterior maxillary segment was passively placed into a predetermined palatal acrylic splint and fixed with posterior teeth by circumdental wiring (Figure 3C). No maxillary intersegmental fixation or maxillomandibular fixation was involved. The palatal splint was removed 6 weeks postoperatively.

**Figure 4:**
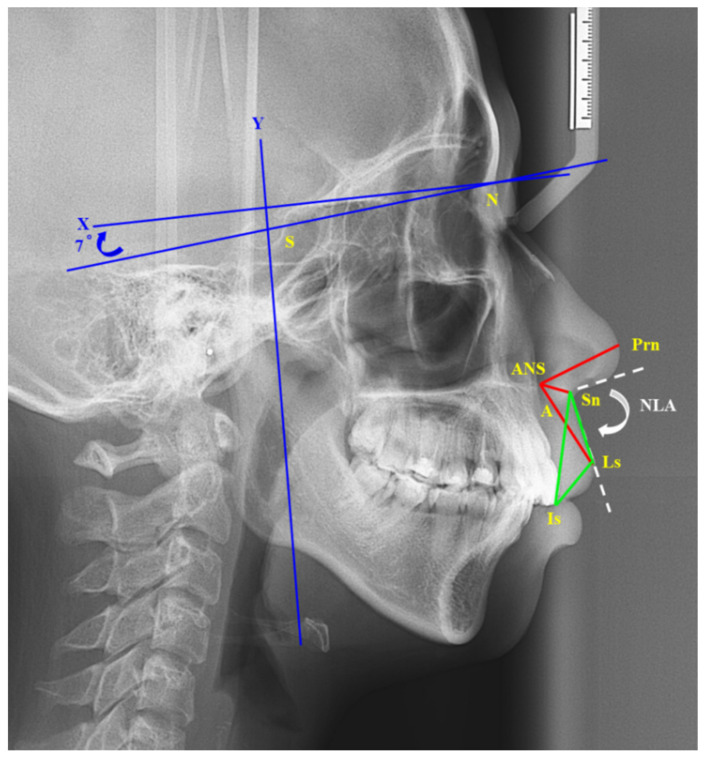
Cephalometric landmarks: sella (S), nasion (N), point A, pronasale (tip of nose, Prn), subnasale (Sn), labrale superius (Ls), anterior nasal spine (ANS), and incisor superius (Is). The X-axis (horizontal line) was constructed by drawing a line through N and 7° above the NS line, and the Y-axis (vertical line) passed through S and was perpendicular to the X-axis. The following distances and angles were measured: red line: ANS–Prn, ANS–Sn, ANS–Ls; green line: Is–Sn, Is–Ls, Ls-Sn; white dotted line: nasolabial (NLA) angle.

For quantifying volumetric changes in postoperative facial edema, van der Vlis et al. [17] used 3D stereophotogrammetry to measure the postoperative swelling after orthognathic surgery. They found that facial edema resolves approximately 80% during the first 2 months. Usually, the patient was referred to the orthodontist to begin orthodontic treatment at the postoperative 2 months. To reduce the effect on the surgical outcome by orthodontic treatment, the cephalograms were obtained before the operation and 2 months postoperatively. The following landmarks (Figure 4) were recorded: sella (S), nasion (N), point A, pronasale (tip of nose, Prn), subnasale (Sn), labrale superius (Ls), ANS, and incisor superius (Is). The *x*-axis (horizontal line) was constructed by drawing a line through N and 7° above the NS line, and the *y*-axis (vertical line) passed through S and was perpendicular to the *x*-axis. The following distances and angles were measured: ANS–Prn, ANS–Sn, ANS–Ls, Is–Sn, Is–Ls, and nasolabial (NLA) angle.

Statistical analyses were processed using IBM SPSS Statistics for Windows, version 20 (IBM Corp., Armonk, NY, USA), and data are presented as means and standard deviations. Statistical significance was determined at a 95% confidence level using the *t*-test. This study protocol was approved by the Human Investigation Review Committee of Kaohsiung Medical University Hospital (KMUHIRB-20140362).

## 3. Results

Sn and Ls were at 11 and 10.5 mm anterior to ANS and Is, respectively (Table 1). Comparing the horizontal hard tissue landmarks before and after the procedure, both Is and ANS were significantly retracted, by 7.3 (*p* < 0.001) and 2.3 mm (*p* < 0.001), respectively (Table 1 and Table 2). In the vertical direction, both Is and ANS moved significantly upward by 2.0 (*p* < 0.001) and 1.0 mm (*p* = 0.021), respectively. In terms of facial appearance, soft tissue landmarks (Prn, Sn, Ls) were retracted significantly (by 1.2, 1.6, and 4.4 mm, respectively), but no significant changes were noted in the vertical direction.

As shown in Table 3, the NLA angle increased significantly by 7.1° (*p* < 0.001). The ANS–Prn and ANS–Sn distances increased significantly by 0.7 (*p* = 0.023) and 1.3 mm (*p* = 0.014), respectively. The ANS–Ls distance did not change significantly, but the Is–Sn distance decreased significantly by 1.3 mm (*p* = 0.016). The Is–Ls and Ls–Sn distances exhibited no significant changes. Regarding the horizontal soft/hard tissue ratios, as displayed in Table 4, Prn/Is and Sn/Is were 0.17 and 0.22, respectively. ANS/Is and Ls/Is were 0.31 and 0.60, respectively, and Prn/ANS and Sn/ANS were 0.54 and 0.72, respectively. Regarding the vertical soft/hard tissue ratios, Prn/Is and Sn/Is were −0.15 and −0.29, respectively. ANS/Is and Ls/Is were 0.51 and −0.32, respectively, and Prn/ANS and Sn/ANS were −0.30 and −0.57, respectively.

## 4. Discussion

In 1927, Wassmund [7] modified the Cohn-Stock [6] method in the form of a bilateral vertical releasing incision at the posterior to the medial interdental papilla of the first premolars and a vertical incision through the mucoperiosteum overlying the ANS. The labial and palatal subperiosteal tunneling approaches were performed for osteotomy completion. The Wassmund [7] method maintains the vasculature of both the labial and palatal flaps. Of all AMSO methods, the Wassmund [7] method best maintains the vascularity of the anterior maxillary segment. However, this tunneling technique made the invisible osteotomy approach only with sensing, especially in palatal osteotomy. More difficulty is encountered in the Wassmund [7] method for removing palatal bone for larger setback of the anterior maxilla.

With the evolution of AMSO, the main surgical approach has changed from a tunneling approach (Cohn-Stock [6] method and Wassmund [7] method) to an open approach (Cupar [8] method: labial down-fracture technique and Wunderer [9] method: palatal up-fracture (out-fracture) technique). In 1954, Cupar [8] made an incision similar to that for the Le Fort I vestibular circumferential incision to enable direct surgical vision. The labial approach facilitates osteotomy from the labial side to the palatal side under direct vision. The vasculature of the palatal flap was kept intact. Therefore, blood supply to the anterior maxillary segment was provided by the palatal flap. The Cupar [8] method (down-fracture) is indicated for moderate setback and superior repositioning of the anterior maxilla.

In 1963, Wunderer [9] maintained a labial mucoperiosteal blood supply and advocated for a transpalatal approach with up-fracture of the anterior maxilla. The main advantages of the Wunderer [9] method are that palatal osteotomy for thicker palates is easy to perform and that a larger amount of anterior maxillary setback can be achieved under direct vision. However, similar to the Wassmund [7] method, the Wunderer [9] method still requires a bilateral vertical releasing incision at the posterior to the medial interdental papilla of the first premolar to facilitate labial osteotomy. The Wunderer [9] method provides a lower blood supply than do the Wassmund [7] and Cupar [8] methods in the perioperative periods. Therefore, the Wunderer [9] method does not require a vertical incision overlying the ANS. This causes difficulty in the mucoperiosteal flap reflection of the mesial inferior portion of the piriform aperture and nasal floor, resulting in nasal mucosal tear. Different from the Wassmund [7] and Wunderer [9] methods, our modified AMSO method maintains two more gingiva of the first premolar through an incision at the posterior to distal interdental papilla of the first premolar, providing a greater supply of blood to the anterior maxilla. Similar to the Wassmund [7] method, a vertical incision is made overlying the ANS to facilitate the reflection of the mesial inferior portion of the piriform aperture and nasal floor. Palatal flap incision and palatal osteotomy are curved anteriorly to reduce the volume of the anterior maxilla and promote hemodynamics. In present study, there is no occurrence of buccal mucosal necrosis, palatal tear, and osteonecrosis of the maxilla. It seems to be superior in wound healing due to buccal mucosal incision line shifting from directly above the osteotomy line. Therefore, our modified AMSO method not only provides direct access but also enhances blood supply to the anterior maxilla.

Because the main concern of patients seeking orthognathic surgical treatment is mostly aesthetic in nature, especially for patients with maxillary protrusion, surgeons predict the postoperative profile and inform patients of potential dentoskeletal changes before performing the procedure. The majority of the present patients’ complaints were maxillary protrusion (lateral view) and a gummy smile (frontal view). The most representative of maxillary protrusion are the positions of ANS and Is. In the present study, Is and ANS were significantly retracted by 7.3 and 2.3 mm, respectively. The amount of ANS setback was approximately 31% of that of Is. The Sn and Ls were set back significantly by 1.6 and 4.4 mm, respectively. In sum, the patients’ profiles were improved substantially.

The varying degree of Ls/Is ratio may be attributable to differences in the amount of maxillary anterior segment setback between the ANS and Is. The Ls/Is ratio ranges from 0.33 to 0.97 in the literature [4,5,18,19]. Lew et al. [4] reported a 0.44:1 ratio of Ls/Is (5.93 mm setback) using the Wunderer [9] method. Park and Huang [5] reported a 0.67:1 ratio of Ls/Is (5.93 mm setback) using the Cupar [8] method. The horizontal osteotomy was modified from the inferior side of the ANS and piriform aperture. According to the change in point A, Seon et al. [18] presented an Ls/Is ratio of 0.52 (point A setback of <4 mm) and an Ls/Is ratio of 0.97 (point A setback of ≥4 mm). We observed an Ls/Is ratio of 0.60 (7.3 mm setback) using the modified AMSO method.

Changes in the NLA region are complex following anterior maxillary retraction with different AMSO methods. Seon et al. [4] reported a Sn/ANS ratio of 0.398 and a Prn/ANS ratio of 0.348 in the group with a point A setback of <4 mm. In the group with a point A setback of ≥4 mm, the ratios of Sn/ANS and Prn/ANS were 0.367 and 0.354, respectively. Bhagat et al. [19] reported a Prn/ANS ratio of 0.52. We observed Sn/ANS and Prn/ANS ratios of 0.72 and 0.54, respectively. Our modified AMSO method did not involve the reflection of the soft tissue overlying the ANS. The muscular attachment between the ANS and Sn was maintained. Therefore, the ANS was set back simultaneously by pulling Sn backward. In the Cupar [8] method, the mucoperiosteal flap reflects the muscular attachment between ANS and Sn. The Sn is passively reattached to the ANS after wound closure. Hence, the potential backward movement of Sn is larger in the modified AMSO method than in the Cupar [8] method.

In general, the Asian population has a flatter facial profile and a more obtuse NLA angle compared with the Caucasian population. Postoperative changes in NLA angle are affected by the AMSO technique and the amount and direction of maxillary setback. Park and Huang [17] reported that the NLA angle increased by 14.1° under the Cupar [8] method. Lew et al. [16] reported that the NLA angle increased by 12.2° using the Wunderer [9] method. Nadkarni [20] observed an 8.9° increase in NLA angle, and Seon et al. [4] noted an 8.5° (point A setback of <4 mm) and 6.6° (point A setback of ≥4 mm) increase. Brown and Guyuron [21] indicated that the ideal NLA angle is 93.9° to 97.3° in male patients and 96.8° to 100.2° in female patients. Therefore, the alar cinch suture could be used to prevent widening of the nasal floor, flattening of the philtrum, and extremely great NLA angle in the Cupar [8] method. In our study, the NLA angle increased significantly by 7.1° (from 94.1° to 101.2°). AMSO must focus not only on the amount of setback and gingival display but also on the NLA angle.

Wu et al. [22] reported that lip thickness was reduced, and philtrum length was increased. Park and Hwang [5] noted that lip thickness was reduced significantly by 2.96 mm, and philtrum length was increased significantly by 2.51 mm. Nadkarni [20] observed that the upper lip was lengthened by 1.0 mm after AMSO. In our study, philtrum length (Ls–Sn) was increased by 1.0 mm, a nonsignificant change. The thickness of the upper (ANS–Sn) and lower portions (Is–Ls) of the lip increased significantly by 1.3 mm and decreased nonsignificantly by 0.2 mm, respectively. This is because the modified AMSO method maintains the attachment of the lip muscle to the ANS, whereas the Cupar [8] method does not.

A gummy smile, characterized by an excessive gingival display, is common in patients with maxillary protrusion. Patients with a gummy smile usually show more than 4 mm of the area from the Is to the lower border of the upper lip when smiling. In a study by Nadkarni [20], teeth display was reduced by 1.46 mm. In our study, Is was significantly intruded by 2.0 mm, and Ls was moved downward by 0.6 mm. Our results revealed that gingival display was reduced by 2.6 mm; the gummy smile was corrected, and the patients’ appearance was improved. Patients with BiP usually show a maxillary protrusion and a gummy smile. Therefore, the anterior maxillary segment must be set back and impacted. The anterior maxillary segment tends to rotate in a clockwise setback, then narrowing the nasal airway space. Therefore, the posterior nasal aspect of the palatal bone should be trimmed to compensate for the reduction in the nasal airway space.

## 5. Conclusions

Our modified AMSO method provides increased blood supply, allows for direct vision, and results in fewer complications than other AMSO methods. Postoperative changes of horizontal soft/hard tissue ratio were 0.17 (Prn/Is), 0.22 (Sn/Is), 0.31 (ANS/Is), and 0.60 (Ls/Is). Prn/ANS and Sn/ANS were 0.54 and 0.72, respectively. The NLA angle was increased significantly by 7.1°.

## Figures and Tables

**Table 1:** Summary of preoperative landmarks (*n* = 33).

Variables	Horizontal (mm)		Vertical (mm)	
	Mean	SD	Mean	SD
Prn	97.9	3.60	49.7	3.02
Sn	84.6	4.55	61.1	3.53
ANS	73.6	3.89	57.2	2.92
Ls	90.7	5.14	77.8	4.10
Is	80.1	6.46	92.4	4.28

*n*: number of patients.

**Table 2:** Summary of surgical change (*n* = 33).

Variables	Horizontal (mm)			Vertical (mm)		
	Mean	SD	*p* Value	Mean	SD	*p* Value
Prn	−1.2	1.51	<0.001 *	0.3	1.62	0.341
Sn	−1.6	2.62	0.001 *	0.6	1.90	0.076
ANS	−2.3	2.01	<0.001 *	−1.0	2.26	0.021 *
Ls	−4.4	3.07	<0.001 *	0.6	2.41	0.158
Is	−7.3	3.78	<0.001 *	−2.0	2.76	<0.001 *

*n*: number of patients. *: Intragroup comparison: Statistically significant, *p* < 0.05.

**Table 3:** Preoperative characteristics (angle and distances) and surgical change (*n* = 33).

Variables			Surgical Change		*p* Value
	Mean	SD	Mean	SD	
NLA (degree)	94.1	12.57	7.1	9.04	<0.001 *
ANS−Prn (mm)	25.6	2.73	0.7	1.79	0.023 *
ANS−Sn (mm)	11.9	3.34	1.3	2.81	0.014 *
ANS−Ls (mm)	27.1	2.42	−0.1	2.64	0.755
Is−Sn (mm)	32.0	3.04	−1.3	2.82	0.016 *
Is−Ls (mm)	18.5	2.56	−0.2	2.57	0.609
Ls−Sn (mm)	16.2	3.27	1.0	4.25	0.190

*n*: number of patients. *: Intragroup comparison: Statistically significant, *p* < 0.05.

**Table 4:** The horizontal and vertical soft/hard tissue ratios.

Variables	Horizontal	Vertical
Prn/Is	0.17	−0.15
Sn/Is	0.22	−0.29
ANS/Is	0.31	0.51
Ls/Is	0.60	−0.32
Prn/ANS	0.54	−0.30
Sn/ANS	0.72	−0.57

## Data Availability

The data that support the findings of this study are available from the corresponding upon reasonable request.
